# Relationship of the Chemokine, CXCL12, to Effects of Dietary Fat on Feeding-Related Behaviors and Hypothalamic Neuropeptide Systems

**DOI:** 10.3389/fnbeh.2016.00051

**Published:** 2016-03-21

**Authors:** Kinning Poon, Jessica R. Barson, Hui T. Ho, Sarah F. Leibowitz

**Affiliations:** Laboratory of Behavioral Neurobiology, The Rockefeller University, New YorkNY, USA

**Keywords:** (C-X-C motif) ligand 12 (CXCL12), high fat diet, hypothalamus, neuropeptide, locomotor activity

## Abstract

The intake of a high fat diet (HFD), in addition to stimulating orexigenic neuropeptides in the hypothalamus while promoting overeating and reducing locomotor behavior, is known to increase inflammatory mediators that modulate neuronal systems in the brain. To understand the involvement of chemokines in the effects of a HFD, we examined in rats whether HFD intake affects a specific chemokine, CXCL12, and its receptors, CXCR4 and CXCR7, in the hypothalamus together with the neuropeptides and whether CXCL12 itself acts similarly to a HFD in stimulating the neuropeptides and altering ingestion and locomotor behavior. Compared to low-fat chow, a HFD for 5 days significantly increased the expression of CXCL12 and its receptors, in both the paraventricular nucleus (PVN) where the neuropeptides enkephalin (ENK) and galanin were also stimulated and the perifornical lateral hypothalamus (PFLH) where orexin (OX) and melanin-concentrating hormone (MCH) were increased. In contrast, the HFD had no impact on expression of CXCL12 or its receptors in the arcuate nucleus (ARC) where the carbohydrate-related peptide, neuropeptide Y (NPY), was suppressed. Analysis of protein levels revealed a similar stimulatory effect of a HFD on CXCL12 levels in the PVN and PFLH, as well as in blood, and an increase in the number of CXCR4-positive cells in the PVN. In the ARC, in contrast, levels of CXCL12 and number of CXCR4-positive cells were too low to measure. When centrally administered, CXCL12 was found to have similar effects to a HFD. Injection of CXCL12 into the third cerebral ventricle immediately anterior to the hypothalamus significantly stimulated the ingestion of a HFD, reduced novelty-induced locomotor activity, and increased expression of ENK in the PVN where the CXCR4 receptors were dense. It had no impact, however, on NPY in the ARC or on OX and MCH in the PFLH where the CXCR4 receptors were not detected. These results, showing CXCL12 in the hypothalamus to be stimulated by a HFD and to mimic the effects of the HFD where its receptors are located, suggest that this chemokine system may have a role in mediating both the neuronal and behavioral effects induced by a fat-rich diet.

## Introduction

The intake of a diet rich in fat has been associated with a higher prevalence of obesity (Bray et al., 1981; Flatt, 1988), which is accompanied by behavioral changes including an increase in ingestive behavior (Warwick et al., 2003; Chang et al., 2004) and a reduction in spontaneous, overall locomotor activity (Barzel et al., 2015; Wong et al., 2015). Studies in the hypothalamus, a brain region involved in controlling food intake, energy balance, and related behaviors, have identified in animals a variety of orexigenic neuropeptides which mediate these behaviors affected by a fat-rich diet. These neuropeptides include enkephalin (ENK) and galanin (GAL) in the hypothalamic paraventricular nucleus (PVN) and also orexin (OX) and melanin-concentrating hormone (MCH) in the perifornical lateral hypothalamus (PFLH). In adult animals, central injection of these neuropeptides or their analogs stimulates preferential intake of a high-fat diet (HFD) (Robert et al., 1989; Kyrkouli et al., 1990a,b; Gomori et al., 2003; Naleid et al., 2007), and HFD intake in turn increases the expression and levels of these orexigenic neuropeptides in the PVN and PFLH (Akabayashi et al., 1994; Elliott et al., 2004; Chang et al., 2007; Gaysinskaya et al., 2007), suggesting that they function within a positive feedback circuit to promote excess consumption. Administration of these neuropeptides also induces changes in locomotor behavior, with subcutaneous or intracerebral injection of an ENK analog found to depress locomotor activity (Bhargava, 1978; Szekely et al., 1980), similar to an effect produced by the consumption of a HFD (Novak et al., 2010; Wong et al., 2015). This positive relationship of these neuropeptides in the PVN and PFLH to dietary fat is very different from that seen in the hypothalamic arcuate nucleus (ARC), where another orexigenic peptide, neuropeptide Y (NPY), is reduced by intake of a HFD and positively associated with ingestion of a carbohydrate-rich diet (Stanley et al., 1985; Jhanwar-Uniyal et al., 1993; Brown et al., 1998). This evidence, closely relating these orexigenic peptides to dietary macronutrients, lead us to question the molecular and cellular mechanisms that may mediate the effects of a HFD on these neuropeptide systems and subsequently on the ingestive and locomotor behaviors they control.

Recent studies have brought attention to neuroinflammatory systems, which affect neurochemical processes and various behaviors. Spontaneous HFD consumption and dietary obesity are often associated with a state of systemic low-grade inflammation, which is known to affect the functioning of peripheral organs (Milanski et al., 2009; Takanabe-Mori et al., 2010) and may also influence the brain. The ingestion of a HFD similar to dietary obesity can induce inflammation in both the peripheral and the central nervous system, which is associated with an increase in the production of neuroimmune factors such as the cytokines, interleukin 1-beta and tumor necrosis factor-alpha, and a superfamily of small proteins called the *chemo*tactic cyto*kines*, also known as chemokines (Barbarroja et al., 2010; Gregor and Hotamisligil, 2011). While chemokines have been suggested to play an important role in systemic obesity (Rull et al., 2010; Gregor and Hotamisligil, 2011), their function specifically in the neural control of consummatory and related behaviors has not been well characterized. There is one particular chemokine, (C-X-C motif) ligand 12 (CXCL12), which has diverse effects on the function of different neuronal cell types in the brain. Specifically, CXCL12 is shown to promote the migration of dopaminergic neurons in the midbrain (Wu et al., 2009; Wang et al., 2011) and modulate electrical excitability of neurons in the hypothalamus (Guyon et al., 2005b; Callewaere et al., 2006; Guyon et al., 2006). This evidence, together with the finding that HFD intake increases the levels of CXCL12 in circulating immune cells (Fenton et al., 2009), hints at the possibility that this chemokine may be involved in the effects of a HFD on brain neuropeptides and certain behaviors.

To test this possibility, the present report examined and compared the effects of a HFD to those of central CXCL12 administration on the CXCL12 and neuropeptide systems in the hypothalamus and on the consumption of a HFD and related behaviors. Specifically, using both molecular and behavioral measures, this study tested whether: (1) endogenous gene expression of CXCL12 and its receptors, CXCR4 or CXCR7, is stimulated by HFD intake, in the same hypothalamic nuclei where the orexigenic neuropeptides are also increased by dietary fat; (2) protein levels of CXCL12 and its receptors are similarly stimulated by HFD intake; (3) central administration of CXCL12, like consumption of a HFD, increases caloric intake and reduces novelty-induced locomotor activity; and (4) central CXCL12 injection also stimulates the expression of orexigenic neuropeptides believed to mediate these behaviors.

## Materials and Methods

### Animals

**FIGURE 1:**
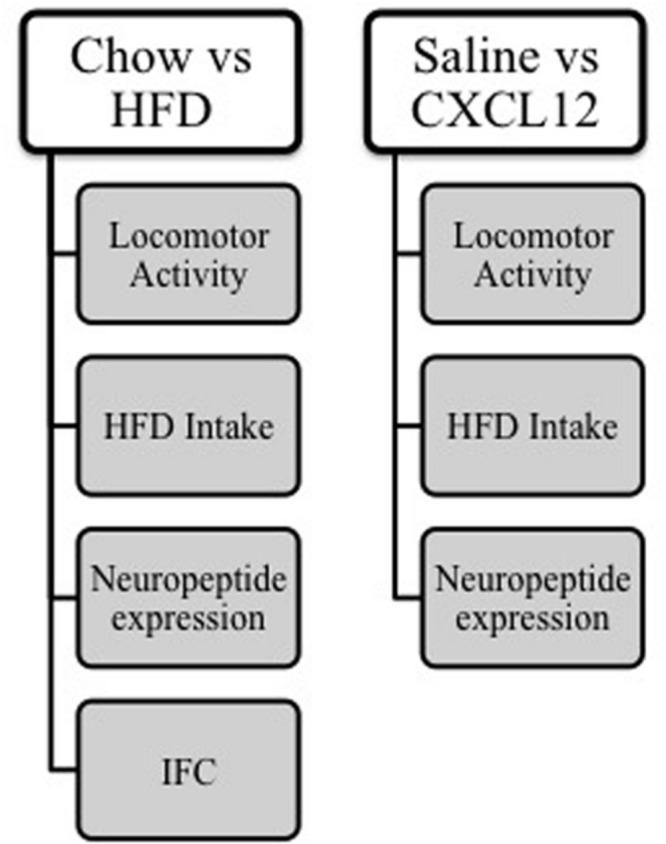
**Schematic of methods.** Four groups of rats were given either chow or HFD (*n* = 16/group) and measurements were taken of locomotor activity, HFD intake, and neuropeptide expression, and immunofluorescence histochemistry was performed. A additional group of rats was cannulated (*n* = 18) and given intracerebral injections of either saline or CXCL12, and measurements were taken of locomotor activity, HFD intake, and neuropeptide expression.

Five groups (**Figure 1**) of adult, male Sprague–Dawley rats (*N* = 82), weighing between 250 and 300 g at the start of all experiments (Charles River Breeding Labs, Kingston, NY, USA), were individually housed (22°C, with lights off at 9:00 a.m. for 12 h) in a fully accredited American Association for the Accreditation of Laboratory Animal Care facility, according to institutionally approved protocols as specified in the National Institutes of Health Guide to the Care and Use of Animals and also with the approval of the Rockefeller University Animal Care and Use Committee. All animals were given 1 week to acclimate to lab conditions, during which time they were maintained *ad libitum* on low-fat laboratory chow (PicoLab Rodent Diet 20 5053, Lab Diet, St. Louis, MO, USA; 12% fat, 60% carbohydrate, and 28% protein) and filtered water. All efforts were made to minimize the use and suffering of animals. The first four groups of rats (*N* = 64, *n* = 16 per group) were used in the HFD experiments, while the fifth group (*N* = 18, *n* = 9 per group) was used in a within-subject design to test the effects of CXCL12 injection, first on novelty-induced locomotor activity, then on acute HFD intake, and finally, on neuropeptide expression.

### Diet

For the experimental period, unless otherwise stated, rats were maintained *ad libitum* on standard rodent chow (12% fat, 3.3 kcal/g). For the four groups of rats tested with HFD or chow (*n* = 8/diet group), the HFD groups were acclimated for 3 days prior to HFD testing with a 15 kcal pellet of the HFD in addition to their regular chow. With the chow then removed, they were given 5 days of 24-hr access to a HFD alone, while the chow groups were given only the laboratory chow diet. This short-term HFD regimen was used to examine the effects of the diet itself while minimizing any changes in metabolism and body weight produced by long-term consumption of a HFD. For the fifth group of rats used in the CXCL12 injection experiments, both HFD and chow were available during intake testing, with the rats exhibiting a strong propensity to eat the HFD over chow. The HFD diet consisted of 50% fat (5.15 kcal/g), as described in our previous publications (Dourmashkin et al., 2006). It had 50% fat composed of 75% lard (Armor Star, Peoria, IL, USA) and 25% vegetable oil (Crisco, Orrville, OH, USA), 25% carbohydrate from 30% dextrin (ICN Pharmaceuticals, Costa Mesa, CA, USA), 30% cornstarch (ICN Pharmaceuticals, Costa Mesa, CA, USA), and 40% sucrose (Domino Foods Inc., Yonkers, NY, USA), and 25% protein from casein (Bio-Serv, Frenchtown, NJ, USA). It was supplemented with minerals (USP XIV Salt Mixture Briggs; ICN Pharmaceuticals, Costa Mesa, CA, USA) and vitamins (Vitamin Diet Fortification Mixture; ICN Pharmaceuticals, Costa Mesa, CA, USA). This diet is nutritionally complete and does not have any detrimental effects on the health of the animals. It was stored at 4°C until use. Caloric intake and body weight were recorded daily.

### Brain Dissections

Rats were sacrificed by rapid decapitation, after 5 days of HFD or chow access in the first and second groups or one hr after CXCL12 injection in the fifth group. All food was removed 1 hr prior to sacrifice. For the microdissections, the brain immediately after sacrifice was placed in a matrix slicing guide with the ventral surface facing up. Four 1.0 mm coronal cuts were made, with the anterior middle optic chiasm (Bregma -1.3 mm) as the anterior boundary (Paxinos and Watson, 2005). The second cut was made 1.0 mm caudal to this, yielding a slice (Bregma -1.3 to -2.3 mm) that was used for microdissection of the PVN. The third cut was made 1.0 mm caudal to the prior cut, yielding a slice (Bregma -2.3 to -3.3 mm) used for microdissection of the PFLH and ARC. These sections were placed on a glass slide and rapidly dissected under a microscope. The PVN was dissected as a reversed isosceles triangle, 1.0 mm bilateral to the third ventricle and between the fornix structures. The PFLH was taken bilaterally from the area surrounding each fornix, with a target range of 0.2 mm medial and ventral to the fornix, 0.3 mm dorsal, and 0.4 mm lateral. The ARC was dissected as an isosceles triangle dorsal to the median eminence, 0.5 mm bilateral to the third ventricle. These dissections were either stored in RNAlater (Sigma–Aldrich) for mRNA extraction or flash frozen in isopentane for protein extraction.

### Quantitative Real-Time Polymerase Chain Reaction (qRT-PCR)

Neuropeptide and chemokine expression was examined at the same time that neuropeptide changes have been previously detected (Chang et al., 2007; Gaysinskaya et al., 2007), from the first and fifth group using qRT-PCR in different, microdissected brain regions. The mRNA from each microdissected sample was purified using a Qiagen RNeasy kit (Qiagen, Valencia, CA, USA), cDNA was synthesized using high capacity RNA-to-cDNA master mix (Life Technologies, Grand Island, NY, USA), and the SYBR Green PCR core reagents kit (Life Technologies, Grand Island, NY, USA) was used for qRT-PCR and was performed in MicroAmp Optic 96-well Reaction Plates (Life Technologies, Grand Island, NY, USA) under the condition of 2 min at 50°C, 10 min at 95°C, and 40 cycles of 15 s at 95°C and 1 min at 60°C, as previously described (Poon et al., 2012). The levels of target gene expression were quantified relative to the level of cyclophilin-A, using the relative quantification method. Primers were designed with the NCBI Primer design tool (http://www.ncbi.nlm.nih.gov/tools/primer-blast/) to span an exon-exon gap to eliminate amplification of genomic DNA. The primers used were: CXCL12 forward: 5′-AGTGACGGTAAGCCAGTCAGCCT-3′, reverse: 5′-TGACGTTGGCTCTGGCGACA-3′; CXCR4 forward: 5′-GGGCTGGAGAGCGAGCATTGC-3′, reverse: 5′-AAGCAGGGTTCCTTGTTGGAGTCA-3′; CXCR7 forward: 5′-GCCGCGAGGTCACTTGGTTG-3′, reverse: 5′-CAGGGCCAGTTGATGTCCGAGTA-3′. The primers for ENK, GAL, ENK, OX, MCH, NPY, and CYC were designed as previously described (Chang et al., 2004). The specificities of PCR products were confirmed by a single band of corresponding molecular weight revealed by agarose gel electrophoresis. The concentration of all target primers was 100 nM, and the CYC primer was 200 nM.

### ELISA

Trunk blood and dissected brain regions were collected from the second group of rats, and protein levels of CXCL12 were measured via ELISA. Tissue was lysed in RIPA lysis buffer (Santa Cruz Biotechnology, Santa Cruz, CA, USA) and homogenized manually using a mortar and pestle. A Lowry assay was performed to determine the total protein in each sample, and afterward, the homogenate was concentrated using a protein concentrator (Millipore, Billerica, MA, USA). A mouse CXCL12 ELISA kit (R&D Systems, Minneapolis, MN, USA) was used to measure levels of hypothalamic CXCL12 and performed according to the manufacturer’s instructions. A total of 0.5 μg of protein was loaded into each well. Protein levels of CXCL12 were calculated relative to the standard curve of the standard samples.

### Immunohistochemistry

Rats in the third group, after being given access for 5 days to a HFD or chow diet, were deeply anesthetized with 20 mg/kg xylazine (LLOYD Incorporated, Shenandoah, IA, USA) and 100 mg/kg ketamine (Fort Dodge Animal Health, Overland Park, KS, USA) (i.p.). They were perfused transcardially with 0.9% NaCl and then 4% paraformaldehyde, as previously described (Barson et al., 2015). Brains were removed, post-fixed in 4% paraformaldehyde for 24 h, transferred to 25% sucrose for 4 days at 4°C, and then frozen at -80°C. Coronal brain slices containing the different regions of the hypothalamus were processed for CXCR4 immunohistochemistry, using a 1:200 dilution of rabbit anti-CXCR4 (Abcam, Cambridge, MA, USA) and a 1:400 dilution of Alexafluor secondary antibody, goat anti-rabbit 594 (Life Technologies, Grand Island, NY, USA). Several antibodies for CXCR7 were tested but did not label properly, suggesting a lack of effective commercially available antibodies for CXCR7. Although protein levels of CXCR7 could not be measured, the existence of high levels of CXCR7 mRNA suggests a possibility that this receptor may be present in these brain regions as shown in transgenic mice (Banisadr et al., 2003).

### Cannulations

Rats in the fifth group were anesthetized with ketamine (75 mg/kg, i.p.) and xylazine (10 mg/kg, i.p.) and supplemented with ketamine when necessary. Stainless steel 21-gage guide shafts (10 mm in length) were implanted in the following coordinates: Posterior: -3.1 mm, Lateral: 0.0 mm, Ventral: -6.0 mm, with reference to bregma, the midsaggital sinus, and the level skull surface. Stainless steel stylets were left in the guide shafts between injections to prevent occlusion. All animals had 1 week to recover before testing, during which time they were handled daily and their stylet was removed and replaced to acclimate them to the injection procedure.

### Microinjections

For the fifth group of rats, CXCL12 was delivered through 26-gage stainless steel microinjectors with fused-silica tubing inside (74 μm ID, 154 μm OD, Polymicro Technologies, Phoenix, AZ, USA) that protruded 3 mm beyond the guide shaft to reach the third cerebral ventricle (icv; Ventral -9.0 mm), where injections could reach the hypothalamic nuclei. The CXCL12 (Prospec-Tany Technogene Ltd, Ness-Ziona, Israel) was dissolved in preservative-free 0.9% saline solution (Hospira Inc., Lake Forest, IL, USA) immediately prior to microinjection, to achieve a final concentration of 50 ng/3 μL, a dose used in other studies to activate other neurochemical systems in the brain and also alter locomotor behavior (Skrzydelski et al., 2007; Trecki and Unterwald, 2009). Injections across the experiments, which measured HFD intake, novelty-induced locomotor activity, and neuropeptide expression, were balanced so that each rat received injections of vehicle or 50 ng CXCL12 at least one time but never twice in a row. Injections were given 3 h into the dark cycle, immediately prior to behavioral testing. A syringe pump delivered 3 μL during 60 s, and microinjectors were left in place for an additional 60 s to allow for diffusion, after which rats were placed in the locomotor activity chamber or in their home cage where they were given a HFD. At least 4 days elapsed between injections to allow for recovery.

### Histological Analysis

**FIGURE 2:**
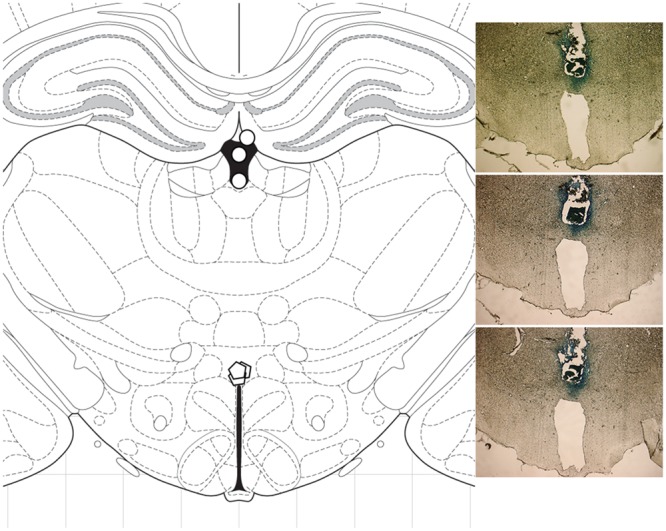
**Histological analysis.** To verify the sites of microinjections, methylene blue was injected into three rats, and the brains were sliced as 30 μm coronal sections. Methylene blue was detected at a 3 mm radial spread from the injection site. **(Left)** diagram of coronal section at -3.12 mm from bregma; ○ cannulation site, ○ injection site. **(Right)** photomicrographs showing cannula placement in the three rats.

To precisely verify the sites of microinjections, three rats were injected with methylene blue dye and then were sacrificed by rapid decapitation. Their brains were fixed and sliced on an electronic microtome cryostat at -25°C as 30 μm coronal sections and examined microscopically. Methylene blue was found at a 3 mm radial spread from the injection site (**Figure 2**). For all rats used in the experiment, verification of the injection sites was performed visually during their brain dissections.

#### Behavioral Testing

Rats in the fifth group that were not exposed to any stimuli and were maintained on chow were tested 3 h into the dark cycle for the effects of CXCL12 injection on novelty-induced locomotor activity during first exposure. They were injected in a between-subject design with 50 ng CXCL12 or with saline and were moved 10 min later from the vivarium to a sound-attenuated room and placed in a 17.0″ × 17.0″ (43.2 cm × 43.2 cm) open field activity test chamber for 15 min (Med Associates, Inc., St. Albans, VT, USA). For comparison with the effects of CXCL12 injection, the rats in the fourth group that were maintained for 5 days on a HFD or chow were given a similar 15-min test in the open field activity chamber. In both groups, ambulatory episodes, ambulatory distance, ambulatory time, and ambulatory counts, which were quantified as the number of infrared beam breaks, were recorded automatically during this 15-min period. Next, to test the effects of CXCL12 on HFD intake, the same rats were first trained to consume a HFD by being given a small, 15-kcal meal of this diet daily for 3 days, with chow also available. Three hours into the dark cycle, the rats were then injected in a within-subject design with 50 ng CXCL12 or with saline, and their intake of HFD was measured at 1, 3, and 24 h after injection.

### Data Analysis

Differences between the effects of CXCL12 at multiple time-points were assessed using a two-way repeated-measures ANOVA, with time as a repeated measures followed up by a one-way ANOVA for each time and then paired, two-tailed *t*-tests when appropriate. Differences between HFD intake were assessed using two-tailed *t*-tests. A one-way ANOVA was used in experiments examining gene expression, followed by Bonferroni’s *post hoc* test when appropriate. Significance was determined at *p* < 0.05. Data are reported as mean ± standard error of the mean (SEM).

## Results

### HFD Intake Increases mRNA Expression of CXCL12 and its Receptors

**FIGURE 3:**
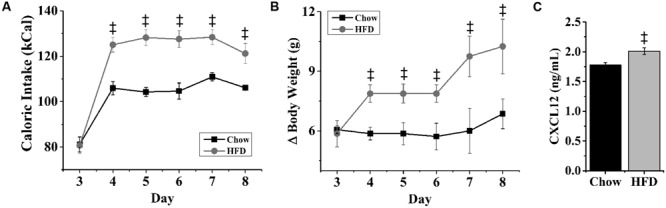
**Effects of high-fat diet on caloric intake, body weight and CXCL12.** Ingestion of a HFD for 5 days significantly increased: **(A)** daily caloric intake, **(B)** body weight, and **(C)** blood levels of CXCL12, as compared to rats ingesting low-fat chow diet. *N* = 16, ^‡^*p* < 0.001.

**FIGURE 4:**
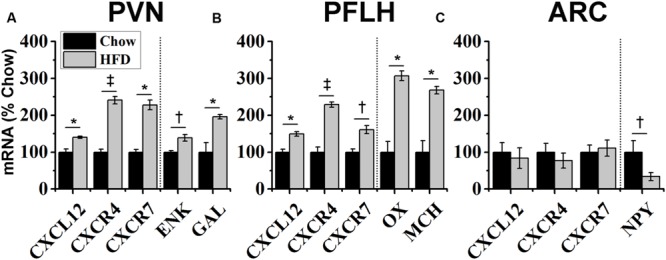
**Effects of high-fat diet on chemokine and neuropeptide expression.** Ingestion of a HFD for 5 days: **(A)** increased the expression of endogenous CXCL12 and its receptors, CXCR4 and CXCR7, in the hypothalamic paraventricular nucleus (PVN), as well as expression of the orexigenic neuropeptides, encephalin (ENK) and galanin (GAL); **(B)** increased the expression of CXCL12 and its receptors in the perifornical lateral hypothalamus (PFLH), as well as expression of the orexigenic neuropeptides, orexin (OX) and melanin-concentrating hormone (MCH); and **(C)** had no effect on CXCL12, CXCR4, or CXCR7 mRNA levels in the arcuate nucleus (ARC), where it reduced expression of the orexigenic, neuropeptide Y (NPY). *N* = 16, ^†^*p* < 0.05, ^∗^*p* < 0.01, ^‡^*p* < 0.001.

With dietary fat known to induce inflammation and stimulate fat-related neuropeptides, this experiment tested whether HFD intake affects endogenous expression of CXCL12 and its receptors, CXCR4 and CXCR7, in the same hypothalamic regions where it affects the orexigenic neuropeptides. Rats consuming the HFD for 5 days compared to those consuming a low-fat chow diet exhibited a significant increase in daily caloric intake (**Figure 3A**) and body weight (**Figure 3B**). In addition, consumption of the HFD significantly increased serum levels of CXCL12 [+13%; *t*(10) = 3.15, *p* < 0.05] (**Figure 3C**). Consistent with prior studies (Dourmashkin et al., 2006), this short-term HFD regimen also had a significant main effect on gene expression across brain areas [*F*(1,4) = 140.47, *p* < 0.001] and a significant diet × gene expression interaction effect [*F*(1,16) = 81.52, *p* < 0.001] (**Figure 4**). In the PVN, measurements of mRNA levels revealed both a main effect of HFD [*F*(1,10) = 1293.50, *p* < 0.001] and an interaction effect between diet × gene expression [*F*(4,20) = 126.80, *p* < 0.001]. These effects in the PVN reflected a significant, HFD-induced increase in the expression of CXCL12 [*t*(10) = 4.27, *p* < 0.01], CXCR4 [*t*(10) = 5.11, *p* < 0.001], and CXCR7 [*t*(10) = 4.21, *p* < 0.01], which was accompanied by an increase in mRNA levels of the orexigenic neuropeptides, ENK [*t*(10) = 2.93, *p* < 0.05] and GAL [*t*(10) = 3.36, *p* < 0.01] (**Figure 4A**). In the PFLH, a main effect of diet [*F*(1,10) = 140.30, *p* < 0.001] and an interaction effect between diet × gene expression [*F*(4,20) = 99.65, *p* < 0.001] were also observed, with the HFD significantly increasing the expression of CXCL12 [*t*(10) = 4.01, *p* < 0.01], CXCR4 [*t*(10) = 6.39, *p* < 0.001], and CXCR7 [*t*(10) = 3.09, *p* < 0.05] in association with a significant increase in expression of the orexigenic peptides, OX [*t*(10) = 4.11, *p* < 0.01] and MCH [*t*(10) = 4.08, *p* < 0.01] (**Figure 4B**). Whereas a main effect of diet [*F*(1,10) = 26.30, *p* < 0.01)] and an interaction effect between diet × gene expression [*F*(4,15) = 12.60, *p* < 0.001] were also observed in the ARC, these effects reflected a very different pattern of change from that seen in the PVN and PFLH. In the ARC, the HFD had no impact on the expression of CXCL12 [*t*(10) = 0.45, *p* = 0.66], CXCR4 [*t*(10) = 1.22, *p* = 0.25], or CXCR7 [*t*(10) = 0.35, *p* = 0.73] and caused a significant decrease in expression of NPY [*t*(10) = 2.41, *p* < 0.05] (**Figure 4C**). These results provide anatomical evidence closely linking changes in the CXCL12 chemokine system to those in the orexigenic neuropeptide systems, with HFD intake stimulating CXCL12 and its receptors in the PVN and PFLH where the fat-related neuropeptides are similarly increased and having no impact on this chemokine or its receptors in the ARC where the carbohydrate-related neuropeptide is suppressed by the HFD.

### HFD Intake Increases Protein Levels of CXCL12 and CXCR4

**FIGURE 5:**
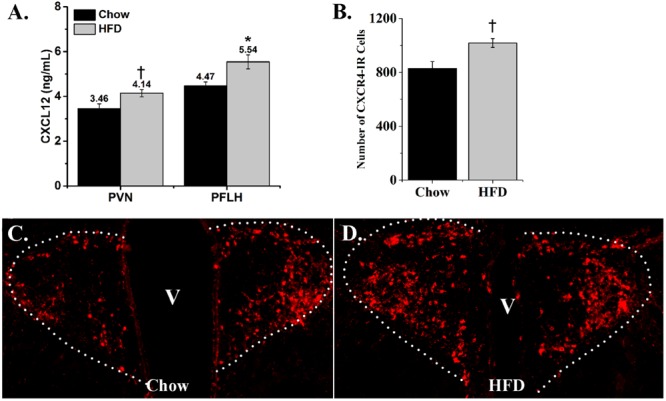
**Effects of high-fat diet on protein levels of CXCL12 and CXCR4. (A)** Ingestion of a HFD for 5 days significantly increased the levels of CXCL12 in both the PVN and PFLH, as compared to rats ingesting low-fat chow diet. *N* = 16. **(B)** Ingestion of a HFD for 5 days significantly increased the number of CXCR4-immunoreactive cells in the PVN, as compared to low-fat chow diet. **(C)** Representative photomicrograph of CXCR4-immunoreactive cells as illustrated in PVN from chow rats; and **(D)** HFD rats. *N* = 16, ^†^*p* < 0.05, ^∗^*p* < 0.01.

With the HFD found to increase mRNA expression of CXCL12 and its receptors in the hypothalamus, this experiment measured whether their protein levels are similarly affected by this diet. Measurements of CXCL12 protein levels in the PVN, PFLH and ARC were performed using an ELISA. The HFD compared to chow diet induced a significant increase in CXCL12 levels in the PVN [+20%; *t*(14) = 2.58, *p* < 0.05] and PFLH [+24%; *t*(14) = 2.99, *p* < 0.01] (**Figure 5A**), while the level of CXCL12 in the ARC was too low to measure. Further analyses of immunofluorescence labeling of CXCR4-positive cells revealed a significant, HFD-induced increase in the density of CXCR4 neurons in the PVN [*t*(8) = 3.08, *p* < 0.05] (**Figure 5B**), as illustrated in the photomicrographs (**Figures 5C,D**). This is in contrast to the ARC as well as the PFLH, where CXCR4-immunoreactive cells were not detected in either the chow or HFD groups. These results show that a HFD increases protein levels, similar to mRNA levels, of CXCL12 and CXCR4 in a site-specific manner, with the PVN showing the strongest and most consistent effect, the PFLH exhibiting a change in CXCL12 but lacking the CXCR4 receptor, and the ARC having undetectable levels of both CXCL12 and CXCR4 under chow and HFD conditions.

### Central Injection of CXCL12 Affects HFD Intake

**FIGURE 6:**
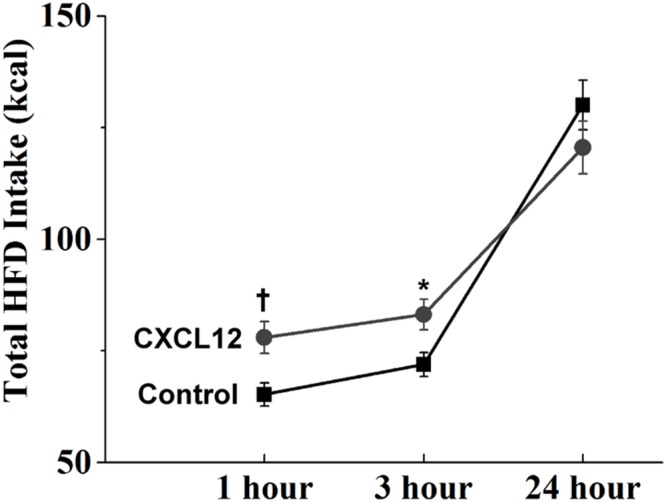
**Effects of CXCL12 on high-fat diet intake.** Injection of CXCL12 into the third ventricle induced a significant increase in HFD intake over the first 3 h post-injection. *N* = 16, ^†^*p* < 0.05, ^∗^*p* < 0.01.

With a HFD generally known to be overconsumed as shown in the first experiment and also found here to stimulate the CXCL12 receptor system, we tested in this next experiment the possibility that central administration of CXCL12 itself compared to saline vehicle acts similarly to a HFD in stimulating caloric intake. This chemokine was injected into the third cerebral ventricle at a dose (50 ng/3 μL) found in other studies to activate other neurochemical systems in the brain and also to alter locomotor behavior (Skrzydelski et al., 2007; Trecki and Unterwald, 2009). At this dose, CXCL12 injection produced a significant change in the intake of a HFD [*F*(1,26) = 1351.87, *p* < 0.001]. This change reflected an increase in caloric intake, which was evident at both 1 h [*t*(52) = 2.89, *p* < 0.01] and 3 h [*t*(52) = 2.57, *p* < 0.05] post-injection but not at 24 h [*t*(52) = 1.18, *p* = 0.24], indicating that ingestive behavior had returned to normal levels (**Figure 6**). While both chow and HFD were present post-injection, the rats solely ate the HFD and not the chow. These results reveal an acute stimulatory effect of CXCL12 itself on HFD intake, suggesting this chemokine may control ingestive behavior.

### Central CXCL12 Injection Is Similar to a HFD in Affecting Novelty-Induced Locomotor Activity

**FIGURE 7:**
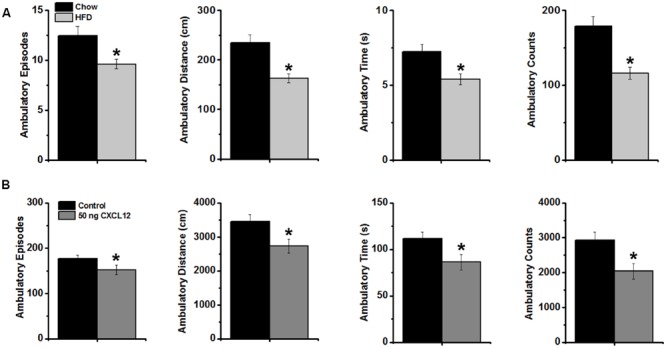
**Effects of a high-fat diet and CXCL12 on novelty-induced locomotor activity. (A)** The intake of a HFD significantly decreased novelty-induced locomotor activity during 15 min in a novel open field, as measured by ambulatory episodes, ambulatory distance, ambulatory time, and ambulatory counts. *N* = 16, ^∗^*p* < 0.01; and **(B)** Intracerebroventricular injection of CXCL12 significantly decreased novelty-induced locomotor activity during 15 min of exposure to the novel open field, as measured by ambulatory episodes, ambulatory distance, ambulatory time, and ambulatory counts. *N* = 16, ^∗^*p* < 0.01.

With CXCL12 found to mimic the stimulatory effect of a HFD on caloric intake and a HFD also known to reduce locomotor behavior (see Introduction), this experiment further tested whether CXCL12 acts similarly to a HFD in affecting novelty-induced locomotor activity in an open field. After 5 days of ingesting HFD compared to chow, measures of locomotor activity, defined as ambulatory episodes, ambulatory distance, ambulatory time, and ambulatory counts, were recorded for 15 min in a novel open field. Analyses of these measures over the full 15 min revealed a significant, main effect of HFD on locomotor activity [*F*(1,56) = 33.20, *p* < 0.001] (Supplementary Table S1). This reflected a significant decrease in this novel environment in the measures of ambulatory episodes, distance, time, and counts (*p* < 0.01) (**Figure 7A**), indicating a reduction in novelty-induced locomotor activity. Similar measurements of locomotor activity for 15 min in a novel open field were then recorded in rats injected in the third cerebral ventricle with saline or CXCL12 at 50 ng. Analyses of these measures over the full 15 min also revealed a significant main effect of CXCL12 on locomotor activity [*F*(1,56) = 16.74, *p* < 0.001), reflecting a significant decrease in measures of ambulatory episodes, distance, time, and counts (*p* < 0.01) (**Figure 7B**). These findings show that CXCL12 is similar to a HFD in causing a decrease in novelty-induced locomotor activity in an open field.

### Central CXCL12 Injection Affects mRNA Expression of Orexigenic Neuropeptides

**FIGURE 8:**
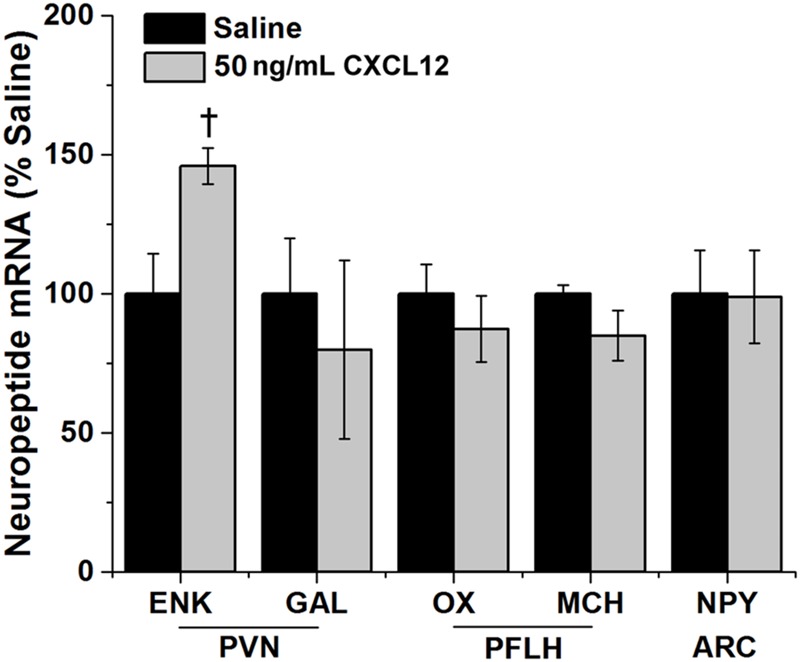
**CXCL12 effects on neuropeptide expression.** Injection of CXCL12 into the third ventricle caused a significant increase in the expression of enkephalin (ENK) in the paraventricular nucleus (PVN), with no effect on galanin (GAL). There was also no effect of CXCL12 on the expression of both orexin (OX) and melanin-concentration hormone (MCH) in the perifornical lateral hypothalamus (PFLH) and on the expression of neuropeptide Y (NPY) in the arcuate nucleus (ARC). *N* = 24, ^†^*p* < 0.05.

This experiment examined whether central injection of CXCL12 can also stimulate the orexigenic neuropeptides believed to be involved in mediating HFD ingestion and novelty-induced locomotor activity. This chemokine was injected into the third cerebral ventricle at 50 ng/3 μL, and its effects on neuropeptide expression in the PVN, PFLH and ARC were measured using qRT-PCR. In the PVN, CXCL12 compared to saline vehicle caused a significant increase in mRNA levels of ENK [*t*(14) == 2.62, *p* < 0.05] (**Figure 8**), similar to the effect produced by a HFD (**Figure 3**). In contrast, CXCL12 injection had no impact on GAL in the PVN [*t*(14) = 0.61, *p* = 0.55], on OX [*t*(14) = 1.61, *p* = 0.13] and MCH [*t*(14) = 1.84, *p* = 0.10] in the PFLH, or on NPY expression in the ARC (**Figure 8**). It also had no impact on its own endogenous system, with mRNA levels of CXCL12 and its receptors, CXCR4 and CXCR7, unaffected in the PVN [*F*(2,14) = 0.40, *p* = 0.68], PFLH [*F*(2,14) = 0.22, *p* = 0.80] and ARC [*F*(2,14) = 0.13, *p* = 0.88]. These results demonstrate a specific stimulatory effect of CXCL12 on the orexigenic peptide ENK in the PVN, where the CXCR4 receptors are dense and stimulated along with this peptide by a HFD, but they reveal no change in OX and MCH peptides in the PFLH or NPY in the ARC where the CXCR4 receptors are not detected.

## Discussion

Although the classical function of chemokines is to induce chemotaxis of immune cells, the current literature suggests a causal relation to HFD intake and a possible role in mediating diet-induced changes in neuronal function and behavior. The results in this study reveal a positive relationship between the CXCL12 system, orexigenic neuropeptides and dietary fat. The ingestion of a HFD stimulates endogenous expression and levels of CXCL12 and its receptors, CXCR4 and CXCR7, in the hypothalamus, and these effects of a HFD occur in precisely the same areas, the PVN and PFLH, where the fat-related orexigenic neuropeptides are also increased by a HFD. This positive relationship is in distinct contrast to our findings in the ARC, where endogenous CXCL12 and its receptors are sparse and unaffected by dietary fat and the neuropeptide NPY is reduced by HFD intake. With central injection of CXCL12, the results further demonstrate that this chemokine has similar effects to a HFD on feeding-related behaviors, causing an increase in caloric intake and a decrease in novelty-induced locomotor activity. Like a HFD, CXCL12 also stimulates expression of the orexigenic peptide ENK in the PVN, where a dense population of CXCR4 receptors are found, but it has no impact on NPY in the ARC where CXCR4 receptors are not detected. Whereas the HFD stimulates the expression of CXCL12 and its receptors in the PFLH where the orexigenic peptides, OX and MCH, are also increased, this hypothalamic area differs from the PVN in having undetectable protein levels of CXCR4 and showing no effect of CXCL12 on the expression of these peptides. These findings indicate that CXCL12 targets specifically the fat-sensitive neuropeptide system in the PVN where it may have a role in mediating the neurochemical and behavioral changes induced by a HFD.

### Region-Specific Effects of a HFD on CXCL12, its Receptors and Orexigenic Neuropeptides

While CXCL12 and its receptors have been identified in various regions of the brain (Banisadr et al., 2003; Rostene et al., 2011), their responsiveness in the brain to a fat-rich diet has yet to be examined. Our results, in addition to confirming a stimulatory effect of a HFD on the orexigenic neuropeptides in the PVN and PFLH (Akabayashi et al., 1994; Elliott et al., 2004; Chang et al., 2007; Gaysinskaya et al., 2007), reveal in these same hypothalamic areas a simultaneous HFD-induced increase in the expression of CXCL12, CXCR4 and CXCR7, and in protein levels of CXCL12, with protein levels of CXCR4 also increased in the PVN but not in the PFLH. Although CXCR7 failed to fluorescently label, the HFD-induced changes in its expression, along with a similar change in expression and protein levels of CXCR4, suggest that protein levels of CXCR7 may also be increased. This close, positive relationship between this CXCL12 system and the fat-stimulated neuropeptides found in the PVN and PFLH is clearly site specific, and not evident in the ARC. In this basal nucleus, the expression and levels of endogenous CXCL12 and its receptors are found to be unaffected by consumption of a HFD, and protein levels of CXCL12 and CXCR4 are undetectable. Also, the orexigenic peptide NPY in this nucleus is reduced by consumption of a HFD, as shown here and in other studies (Giraudo et al., 1994; Wang et al., 1998, 2002), and is positively associated with consumption of carbohydrate rather than fat (Stanley et al., 1985; Jhanwar-Uniyal et al., 1993; Wang et al., 1999). While several other neuropeptides related to ingestive behavior exist in the hypothalamus, the results clearly dissociate this chemokine system from the carbohydrate-related neuropeptide, NPY, in the ARC, in distinct contrast to its positive association with the fat-related neuropeptides in the PVN and PFLH, as identified here.

### Effect of Central CXCL12 Injection on HFD Consumption

With a HFD known to promote excess consumption and also shown to induce a low grade inflammation as confirmed here by elevating circulating levels of CXCL12 during short-term exposure, we examined whether the chemokine, CXCL12, itself can also stimulate the intake of a HFD. Our results show that an acute icv injection of CXCL12 increases HFD consumption for the first 3 h after injection, with intake returning to normal over a 24-h period after injection. Other studies, showing this chemokine to affect expression and levels of other neuromodulators such as MCH and vasopressin in the hypothalamus (Guyon et al., 2005b, 2006; Callewaere et al., 2006) and dopamine in the substantia nigra (Skrzydelski et al., 2007), suggest a broad function of this chemokine on the brain. While HFD itself induces an increase in CXCL12, the results here reveal that an acute central dose of this chemokine similarly effects ingestion, together with changes in orexigenic peptides as described below.

### Similar Effect of HFD and Central CXCL12 Injection on Novelty-Induced Locomotor Behavior

With the ingestion of a HFD known to reduce locomotor behavior (Barzel et al., 2015; Wong et al., 2015) and our results revealing molecular and behavioral effects of CXCL12 that are similar to those of a HFD, we investigated whether this chemokine also affects locomotor behavior in a manner similar to a HFD. The results of the present study support this idea, confirming that intake of a HFD for 5 days reduces locomotor activity in a novel open field, as previously shown (Barzel et al., 2015; Wong et al., 2015), and also demonstrates that central administration of CXCL12 similarly decreases novelty-induced locomotor activity. Other studies have found CXCL12 in various brain regions to modulate locomotor behavior, causing a reduction in activity with injection in the nucleus accumbens (Trecki and Unterwald, 2009) and circling behavior with injection in the substantia nigra (Skrzydelski et al., 2007), once again providing evidence that this chemokine in the brain may have broad effects on behavior. The new results reported here support this idea, showing that CXCL12 in addition to stimulating consummatory behavior also mimics the effects of a HFD on novelty-induced locomotor activity

### Region-Specific Effects of Central CXCL12 Injection on Orexigenic Neuropeptides

With the HFD stimulating CXCL12 in conjunction with neuropeptides in the PVN and PFLH and CXCL12 known to affect the functioning of neurons (Guyon et al., 2005a, 2006; Callewaere et al., 2006; Wu et al., 2009; Wang et al., 2011), it is possible that this chemokine can modulate the hypothalamic peptide neurons involved in ingestion and related behaviors. The results of the present study are consistent with this possibility. They demonstrate that central injection of CXCL12 has a distinct, site-specific effect on ENK in the PVN, a nucleus with a dense concentration of CXCR4 receptors and a neuropeptide that is stimulated by HFD intake (Chang et al., 2007; Gaysinskaya et al., 2007) and preferentially increases intake of a HFD (Robert et al., 1989; Naleid et al., 2007). Thus, CXCL12 when elevated by a HFD may have a role in mediating this diet’s effect on ENK levels and ingestive behavior, and it may also act through ENK to reduce novelty-induced locomotor activity, with central or peripheral injection of this peptide or its analogs shown to decrease locomotor activity (Bhargava, 1978; Szekely et al., 1980). Whereas the ARC is clearly different from the PVN in providing no evidence for a positive relationship between CXCL12, the orexigenic peptide NPY, and dietary fat, the PFLH is similar to the PVN in showing a stimulatory effect of a HFD on the expression of CXCL12, its receptors, and the orexigenic peptides, OX and MCH. However, it clearly differs markedly in revealing no evidence for a role of CXCL12 in mediating the effects of a HFD, with protein levels of CXCR4 undetectable in the PFLH and central injection of CXCL12 having no effect on expression of OX and MCH. While this evidence indicates that HFD-induced stimulation of these neuropeptides in the PFLH occurs independently of the CXCL12 system, other chemokines may be involved. This is consistent with our analyses of GAL in the PVN, a neuropeptide that is positively related to a HFD and increased in a state of dietary obesity (Kyrkouli et al., 1990b; Chen et al., 2005; Inouye et al., 2007) and, while unaffected by CXCL12 as shown here, is stimulated by another chemokine, CCL2 (Poon et al., 2014). Thus, multiple chemokine systems are likely to function together in mediating the effects of a HFD on the different orexigenic neuropeptides and the behaviors they control.

## Conclusion

This study is the first to reveal a site-specific, stimulatory effect of short-term HFD intake on the CXCL12 chemokine system and supports the idea that CXCL12 in the PVN, acting through CXCR4, mimics the molecular and behavioral effects of HFD. The results of this study raise several interesting questions needing further investigation, for example, about the nature of and differences between the CXCR4 and CXCR7 receptors, how inflammation itself might affect the function of neurons, and whether the obese state associated with long-term HFD consumption differentially affects this chemokine system Whereas there are other chemokines possibly involved in mediating the fat-induced changes in neuronal function and behavior, CXCL12, which is known to be abundant in the hypothalamus, is identified here to be similar to a HFD in stimulating HFD intake, orexigenic neuropeptide expression, and feeding-related behaviors.

## Author Contributions

Participated in research design: KP and SL. Performed surgical cannulations and perfusions: JB and KP. Performed and analyzed qRT-PCR data: HH and KP. Conducted and analyzed IFC experiments: KP. Performed and analyzed ELISA: HH and KP. Statistical analysis: KP. Wrote or contributed to writing paper: KP and SL.

## Conflict of Interest Statement

The authors declare that the research was conducted in the absence of any commercial or financial relationships that could be construed as a potential conflict of interest. The reviewer AN and handling Editor declared their shared affiliation, and the handling Editor states that the process nevertheless met the standards of a fair and objective review. The reviewer DC and handling Editor declared their shared affiliation, and the handling Editor states that the process nevertheless met the standards of a fair and objective review.
