# Supplementary material for: Relationship of the Chemokine, CXCL12, to Effects of Dietary Fat on Feeding-Related Behaviors and Hypothalamic Neuropeptide Systems
Source: Front Behav Neurosci. 2016 Mar 21;10:51. doi: 10.3389/fnbeh.2016.00051 (PMC4800166; doi:10.3389/fnbeh.2016.00051)
Supplement: Supplementary file 1 [file Table_1.DOCX]

**Supplemental Data**

| **A.** | **Ambulatory Episodes** | **Ambulatory Distance** | **Ambulatory Time** | **Ambulatory Counts** |
| --- | --- | --- | --- | --- |
| Chow | 12.44 ± 0.96 | 234.76 ± 16.22 | 7.23 ± 0.51 | 179 ± 13.61 |
| ***HFD*** | ****9.63 ± 0.47*** | ****163.21 ± 9.18*** | ****5.40 ± 0.37*** | ****116 ± 8.17*** |
| **B.** |  |  |  |  |
|  | **Ambulatory Episodes** | **Ambulatory Distance** | **Ambulatory Time** | **Ambulatory Counts** |
| Saline | 177.25 ± 8.29 | 3455.88 ± 207.23 | 111.35 ± 7.44 | 2931.38 ± 232.82 |
| 50 ng | 152.43 ± 10.38 | 2732.71 ± 201.53 | 86.24 ± 8.36 | 2042.43 ± 222.48 |

**Supplemental Table 1.** A) Average locomotor activity induced by the intake of a HFD compared to chow over 15 minutes in a novel open field. B) Average locomotor activity induced by intracerebroventricular injection of saline or CXCL12 over 15 minutes in a novel open field.
